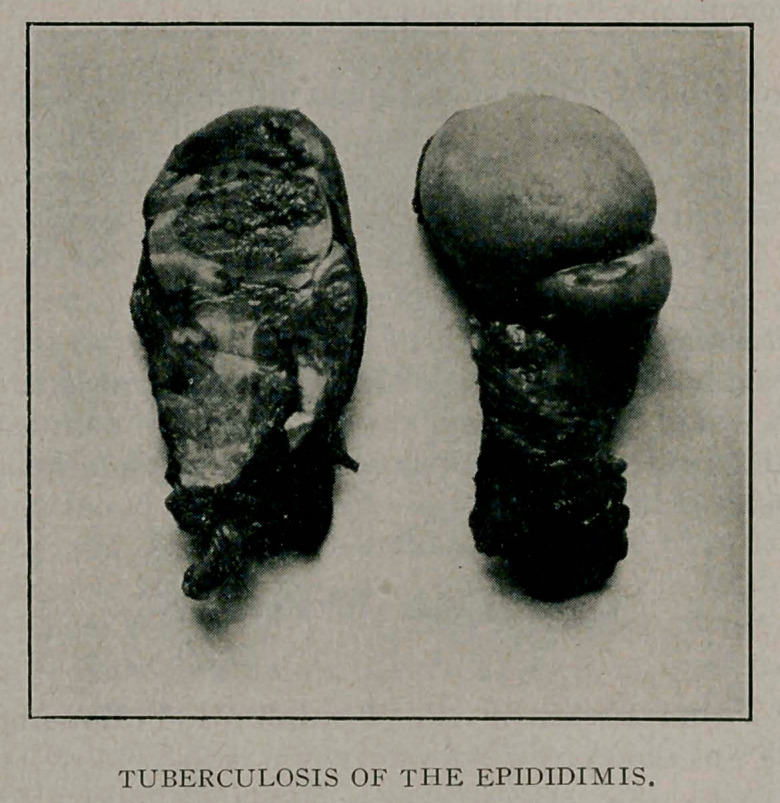# Primary Tuberculosis of the Epididimis Following Injury

**Published:** 1911-01

**Authors:** Charles W. Bethune

**Affiliations:** Buffalo, N. Y.


### CLINICAL REPORTS

---

### Primary Tuberculosis of the Epididimis Following Injury.

By CHARLES W. BETHUNE, M.D., Buffalo, N. Y.

LAST June this patient was referred to me by his family physician for diagnosis and treatment. The history was as follows: nationality, Hungarian; age, 45; married, wife never pregnant; occupation, cabinet maker; denies venereal history; could give no information as to family history; never ill before. Three months ago while splitting fire wood, a cord wood stick flew up and hit him in the genital region; the pain caused by this blow was not severe enough to interrupt his work. Shortly after this he noticed that his right testicle began to enlarge.

Status presens: the patient is anemic but fairly nourished, hemoglobin, 70 per cent.; appetite poor. Apparently the right testicle is enlarged to the size of a goose egg. There is a suggestion of fluctuation when the mass is palpated but the impression is that of soft tissue confined under tension rather than that of fluid. Transillumination negative. At the upper pole of the mass a very hard nodule can be felt; cord normal. A single indurated lymph node is palpable in the right groin above Poupart's ligament. Urinary and sexual symptoms absent. Prostate and vesicles feel normal.

Encephaloid carcinoma of the testicle being suspected the patient was sent to Riverside Hospital and operated upon two days later. The scrotum was opened, the mass delivered and incised. It proved to be a hydrocele, the sac, nearly one-quarter of an inch in thickness, containing straw colored fluid under great tension. The globus major of the epididimis was replaced

TUBERCULOSIS OF THE EPIDIDIMIS.

by a hard pyramidal mass which, upon incision, was seen to be composed of a dense white tissue practically bloodless and penetrating into the glandular substance of the testicle. The cord was pulled upon, ligated and divided as high up as possible. The entire mass, including the sac of the hydrocele, was then removed and the incision in the scrotum closed by a continuous suture. The enlarged lymph node was removed through an incision in the groin and a careful search made through the incision for other nodes. None being found this was also sutured.

The patient made an uneventful recovery, leaving the hospital in seven days. A small sinus persisted at the upper angle of the wound but this closed after two weeks' treatment with bismuth paste.

Microscopic examination demonstrated tuberculosis, many giant cells and extensive caseation being present. The patient continually improved after the operation, his weight increased and the hemoglobin soon reached 100 per cent.

Time alone will tell whether this case was operated upon early enough to entirely remove all the infected tissue.

262 Niagara Street.

## Figures and Tables

**Figure f1:**